# Dysfunction of inhibitory–excitatory cortical circuits induces density-dependent facilitation of motor-evoked potentials after subarachnoid hemorrhage

**DOI:** 10.3389/fneur.2026.1690241

**Published:** 2026-04-01

**Authors:** Minoru Fujiki, Kaito Arimatsu, Hiroyuki Matsuta, Hirotaka Fudaba, Kouhei Onishi, Yukari Kawasaki, Nobuhiro Hata, Mitsuhiro Anan

**Affiliations:** Department of Neurosurgery, School of Medicine, Oita University, Hasama-machi, Oita, Japan

**Keywords:** cortical dysfunction, hematoma density, magnetic stimulation, motor-evoked potentials, subarachnoid hemorrhage

## Abstract

Intraoperative monitoring of motor-evoked potentials (MEPs) is important for preventing serious ischemic complications during aneurysm surgery following subarachnoid hemorrhage (SAH). Reduced intraoperative MEP amplitude indicates corticospinal tract neuronal damage, which should be avoided during surgical maneuvers. We aimed to investigate whether the MEP amplitude immediately before surgery can serve as both a control value for intraoperative MEP and an indicator of the pathophysiology of cortical excitability and inhibitory–facilitatory circuits immediately after SAH and neurological functional disturbance during the perioperative period. The present study focused on the MEP amplitude prior to intraoperative clipping and coil embolization and found that the MEP amplitude in the hemisphere with more significant SAH was significantly higher than that in the contralateral hemisphere. We also demonstrated that the MEP amplitude facilitation ratio significantly and linearly correlated with the initial computed tomography (CT) hematoma density [Hounsfield units (HU)] (*p* < 0.0001). The MEP facilitation ratio linearly correlated with abnormalities in short-interval intracortical inhibition (SICI) and intracortical facilitation (ICF) values. Significant correlations were observed in the SICI and ICF groups with delayed cerebral ischemia (*p* < 0.0001), whereas the ICF group showed a similar trend but weaker correlation (*p* = 0.0732). A high MEP amplitude just before surgery correlates well with Sylvian fissure HU values on initial CT and may reflect dysfunction in the cortical electrophysiological intracortical inhibitory–facilitatory neuronal circuits of SAH.

## 1 Introduction

Infarction due to circulatory disturbance of the major arteries or perforating branches is a serious complication that must be avoided during cerebral aneurysm surgery. Intraoperative motor-evoked potentials (MEPs) are an electrophysiological index that sensitively reflects the activity of corticospinal tract neurons (1–3). Intraoperative MEPs are used to avoid manipulation during surgery, which results in corticospinal neuronal damage. An amplitude reduction of >50% in the control waveform is often used as an alert point for postoperative motor dysfunctions (1, 2).

Thus, the MEP amplitude reduction induced by cerebral ischemic insult related to manipulation during surgery has been the focus of clinical interest.

Conversely, postoperative neurological deterioration is caused by multiple complicated factors, such as symptomatic vasospasms, elevated intracranial pressure, microcirculatory-venous circulatory disturbances, and whole-brain electrophysiological abnormalities (1, 2, 4). Therefore, indicators that clearly reflect the pathophysiological conditions in the immediate and perioperative periods after subarachnoid hemorrhage (SAH) are required.

The Hounsfield unit (HU) value reflects the initial computed tomography (CT) hematoma density in SAH and is an accurate and reliable predictor of symptomatic vasospasm or delayed cerebral ischemia (DCI) (4). Specifically, HU values > 50–60 significantly increase the incidence of symptomatic vasospasm and are widely used in the postoperative management of SAH. SAH due to ruptured cerebral aneurysms can lead to delayed ischemic neuronal damage or DCI within a few days to 2 weeks after onset. Until the 1990s, cerebral vasospasm was believed to cause this phenomenon, and symptomatic cerebral vasospasm was considered synonymous with DCI. However, recent studies have shown that symptomatic vasospasm accounts for approximately ≤ 50% of DCI (5). Furthermore, these studies also suggest that DCI is caused by a combination of multiple factors, such as delayed brain injury after SAH, including a complex of other conditions, such as early brain injury (EBI), cortical spreading depolarization/depression (CSD), microcirculatory disturbance, and venous vascular flow disturbance, in addition to symptomatic vasospasm.

SAH induces the release of glutamate, the principal neurotransmitter in the central nervous system. Excessive glutamate levels can result in serious neurotoxicity. CSD is caused by cortical damage resulting from SAH. This induces neuronal and glial cell depolarization and microcirculatory disturbance due to the propagation of neuronal depolarization through the gray matter via the spreading of K^+^ and glutamate (5, 6). Therefore, it is clinically significant to discuss this pathophysiology based on electrophysiological indices that reflect whole-brain function rather than focusing solely on post-onset main artery constriction and narrowing. This perspective is essential for postoperative management and the development of novel DCI-targeted therapies.

However, the laterality and facilitation of MEP amplitudes immediately before surgery have never been discussed as predictors of DCI in relation to the pathophysiological conditions after SAH.

Furthermore, the precise cortical excitability in the SAH-dominant hemisphere vs. the contralateral hemisphere has never been explored.

Therefore, the present study aimed to examine the pathophysiology of cortical excitability and inhibitory and excitatory circuits in relation to the dominance of hematoma density (i.e., HU values) after SAH.

## 2 Materials and methods

### 2.1 Participants

This study examined 179 patients with cerebral aneurysms who underwent clipping or coil embolization under transcranial electrical stimulation MEP monitoring between January 2007 and July 2014 (59 with internal carotid artery aneurysms, 36 with middle cerebral artery aneurysms, and 84 with other types) (Table 1). All patients were right-handed prior to SAH onset. We evaluated cortical excitability in 10 age-matched healthy adults (mean age of 63.2 ± 3.9 years; range of 58–71 years; five males aged 63.4 ± 4.3; five females aged 63.1 ± 3.6; all right-handed) using transcranial magnetic stimulation (TMS) as a control group. After this study received approval from the Institutional Review Board of Oita University (Approval number: 737), we retrospectively reviewed the medical records of patients hospitalized for SAH during the study period. This study included only patients hospitalized within 24 h of symptom onset with an identified aneurysm as the cause of SAH. All aneurysms were diagnosed using CT angiography or digital subtraction angiography. All patients underwent neck clipping or coil embolization of the aneurysms. Patients who received definitive treatment during the chronic phase (>14 days after the onset of symptoms), those who did not receive definitive treatment, those with an intracranial hematoma accompanied by paralysis, and those who died within 7 days were excluded. After neck clipping or coil embolization, all patients were managed in the intensive care unit for at least 14 days, during which time, changes in neurological findings were carefully observed. If neurological findings worsened, cerebral artery evaluation was performed using digital subtraction angiography or CT angiography. Rho kinase inhibitors (fasudil hydrochloride, 60 mg/day) were administered to all the patients between days 4 and 14. Even if the neurological findings were stable, digital subtraction angiography or CT angiography was routinely performed between days 7 and 11 after symptom onset. Cerebrospinal fluid was managed using lumbar drainage until day 14.

**Table 1:** Characteristics of patients.

		Total	Non-DCI	DCI	*P*-Value
		179	131	48	
Age			65.0 (29–86)	65.5 (35–87)	0.89
Gender					0.72
	Male	55	42	13	
	Female	124	89	35	
Aneurysm location			number (%)		0.21
ACA		8	4 (3.0)	4 (8.7)	
Acom		42	27 (20.3)	15 (32.6)	
BA		9	8 (6.0)	1 (2.2)	
ICA		59	41 (32.3)	18 (34.8)	
MCA		36	27 (20.3)	9 (19.6)	
VA		25	24 (18.0)	1 (2.2)	
Hunt and Kosnik grade					0.86
	1	11	8	3	
	1a	1	1	0	
	2	77	57	20	
	3	34	22	12	
	4	33	27	6	
	5	23	16	7	
WFNS grade					0.17
	1	42	36	6	
	2	61	37	24	
	3	10	8	2	
	4	36	27	9	
	5	30	23	7	
Fisher grade					0.18
	1	2	2	0	
	2	11	11	0	
	3	125	95	40	
	4	23	23	8	
					0.023
	1 and 2	13	13	2	
	3 and 4	166	118	48	
Aneurysm treatment					0.066
	Clipping	135	94 (71.8)	41 (85.4)	
	Coiling	44	37 (28.2)	7 (14.6)	
Prognosis					0.31
	mRS (0–2)	78	61 (46.6)	17 (35.4)	
	mRS (3–6)	101	70 (53.4)	31 (64.6)	

ACA, indicates anterior cerebral artery; Acom, Anterior communicating artery; BA, basilar artery; IC, internal carotid artery; MCA, middle cerebral artery; mRS, modified Rankin Scale; SAH, subarachnoid hemorrhage; Surgeons. VA, vertebral artery; and WFNS, World Federation of Neurosurgical.

### 2.2 Evaluation of the Hounsfield unit (HU) values of the right and left Sylvian fissures and interpeduncular cisterns

**Figure 1:**
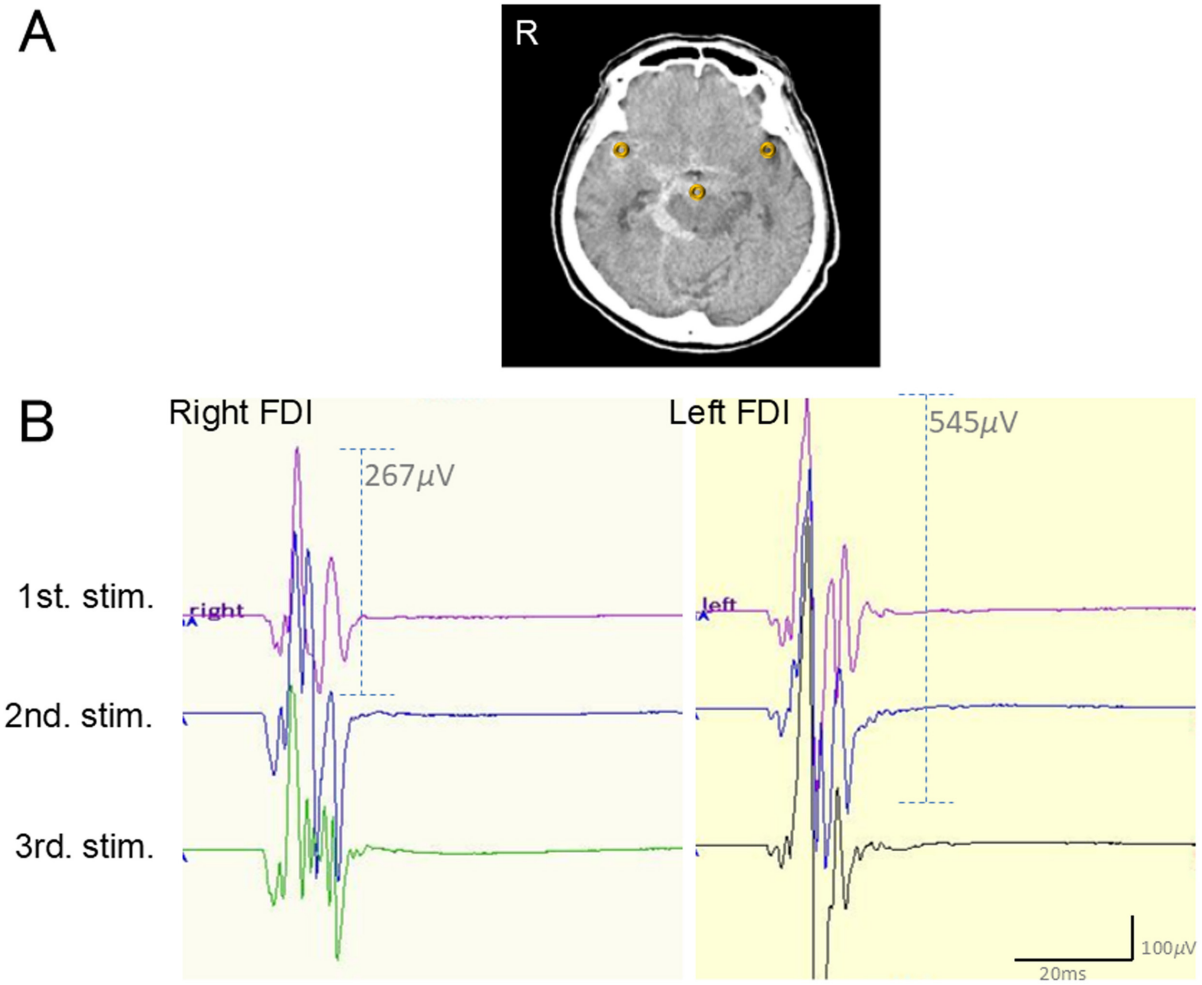
Representative case of a preoperative subarachnoid hemorrhage (ruptured right, internal carotid-posterior communicating aneurysm, 86-year-old, female patient). **(A)** Preoperative computed tomography (CT) scan revealed a dominant subarachnoid hematoma. Circles represent potions that measured Hounsfield unit (HU) values in the right Sylvian fissure, interpeduncular cistern, and left Sylvian fissure (70.37, 45.33, and 35.18, respectively). The right side of the brain in the CT slice is designated as R. **(B)** The motor-evoked potential (MEP) amplitude (left first dorsal interosseous, 545 μV) of the hemisphere with the higher HU value of the Sylvian fissure (right HU value, 70.37) was higher than that of the contralateral side (left HU value, 35.18; right MEP, 267 μV). Control MEP reproducibility was confirmed by serially recording every 1 min (purple: first recording, blue: second recording, green: third recording following first, second and third brain stimulation, respectively).

The evaluation of HU values was based on the method described by Ishihara et al. (4). Briefly, HU values were determined using horizontal CT images with 7.2-mm slices that included the orbital floor. Regions of interest (ROIs) were defined for the interpeduncular cistern and the left and right Sylvian fissures. An 8-mm-diameter circle containing only the hematoma was used as the ROI (Figure 1A). For the analysis of the lateralization of motor cortical excitability, HU values adjacent to the motor cortex and the central sulcus were sometimes difficult to ascertain, and it was challenging to ensure that the ROI contained only the hematoma and no surrounding tissue. Therefore, ROIs for comparing and analyzing the lateralization of motor cortical excitability were chosen from the bilateral Sylvian fissures in the same slice that included the basal and interpeduncular cisterns (7, 8). Two blinded experts (MF and KA) evaluated the mean HU values from the three ROI measurements.

### 2.3 Intraoperative MEP recording

#### 2.3.1 Anesthesia

Anesthesia was induced with propofol (1.5–2 mg/kg) and fentanyl (2 μg/kg) intravenously and maintained with propofol (6–10 mg/kg/h). Fentanyl (2 μg/kg) was added every hour. All patients were administered general venous anesthesia with propofol. No inhaled anesthetics were used in this study.

#### 2.3.2 MEP

The central sulcus line was mapped onto the scalp. A point 7 cm lateral to the midline on this line corresponds to the motor area of the finger. Corkscrew electrodes were placed at points C3 and C4 of the International 10–20 System, and biphasic electrical stimulation was performed. As no case showed a threshold difference exceeding 1mA after measuring the stimulation threshold for each hemisphere, simultaneous bilateral stimulation was performed with a train of five pulses at 120% of the higher threshold (maximum stimulation intensity: 200 mA) (1, 2). Because the transcranial MEP stimulation threshold fluctuates intraoperatively, it was examined once every 10 min. In addition, the threshold was reconfirmed immediately before blocking or clipping maneuvers. A pair of needle electrodes was inserted into the first dorsal interosseous (FDI) muscle contralateral to the stimulus for recording. The filters were set at 20–3,000 Hz. The maximum amplitude of the evoked electromyography (EMG) was monitored, and a warning was issued when a reproducible decrease in amplitude was observed. After temporary blockage of blood flow or clipping, recordings were recorded once per minute and monitored for a minimum of 20 min.

### 2.4 Navigated magnetic stimulation of the motor cortex and MEP recording in patients with SAH

In accordance with previous studies, we applied clinical exclusion criteria for patients with clinical symptoms that would prevent them from undergoing a TMS examination at the time of SAH, such as evidence of seizure or deep coma (9–15). No exception cases were reported during the mean 2.5-day (range 0–3 days) preoperative TMS study period. Before surgery, a frameless stereotaxic brain fixation system combined with a navigated brain stimulation system (NBS; Nexstim eXima, Nexstim Ltd., Helsinki, Finland) was used to guide the coil placement at the bedside (9–15). Both single- and paired-pulse TMS were applied to the primary motor cortex (M1) in the right and left hemispheres using a figure-of-eight coil connected to an EMG amplifier (Neuromaster; Nihon Kohden Co., Ltd., Tokyo, Japan).

Single-pulse TMS was used to determine resting motor threshold (RMT), which is defined as the lowest stimulus that elicits an MEP of at least 50 μV in five out of ten consecutive trials. With the conditioning stimulus set at 80% of RMT and the test stimulus at 120% of RMT, paired-pulse TMS was applied, and short-interval intracortical inhibition (SICI) was measured by taking the ratio of the amplitudes of the MEP response curves at 2-ms and 4-ms inter-stimulus intervals (ISIs). Intracortical facilitation (ICF) was measured by taking the ratio of the amplitudes of the MEP response curves at 10-ms and 15-ms ISIs calculated for each hemisphere by taking the ratio of the amplitudes (11–14).

### 2.5 Definition of outcomes and analysis

In accordance with Ishihara et al. (4), symptomatic vasospasm was defined according to the following criteria: (i) neurological deterioration, (ii) head CT scan ruling out other causes, (iii) no other causes, and (iv) angiographic vasospasm or new infarction. Clinical outcomes were assessed at the 1-month follow-up using the modified Rankin Scale (mRS) in the same manner.

#### 2.5.1 Data analysis

The MEP data were analyzed offline, as previously described (11, 12, 16). All data are presented as the means ± standard deviations, and the level of statistical significance was set at *p* < 0.05. Differences between clinical characteristics in cases with or without DCI were evaluated by Mann-Whitney U test. Categorical variables are shown as frequency counts, and intergroup comparisons were analyzed by Fisher exact test.

For multiple comparisons among different conditions, RMT, MEP amplitudes, SICI, and ICF were analyzed using one-way repeated-measures analyses of variance (ANOVA) with a group as a between subject factor. *Post-hoc* Bonferroni corrections adjusting the significance threshold (α_adj = 0.05/number of comparisons) were also employed in cases of multiple comparison. Correlations between the HU values and MEP amplitudes, SICI, or ICF were evaluated using correlation coefficients (r) and coefficients of determination (R^2^). Analyses were performed using the SPSS software (version 25, IBM Corp., Armonk, NY) and the R software (version 4.3.1, meta package).

From the ANOVA output, the between-group sum of squares, within-group sum of squares, degrees of freedom, mean squares, *F* value, and *p* value were obtained. Effect size η^2^ was calculated using the between-group and total sums of squares. Cohen's f, a standard effect size measure for ANOVA was calculated. *Post-hoc* multiple comparisons were conducted using Bonferroni's test. For each pairwise comparison, Cohen's d was calculated using the mean difference and pooled standard deviation derived from the group means, standard deviations, and sample sizes obtained from the descriptive statistics. This allowed the quantitative evaluation of effect sizes for each pairwise contrast in addition to the overall ANOVA effect.

To assess the influence of outliers on the correlation analysis, we conducted a series of procedures using R software. First, scatterplots were generated to visually inspect the distribution of data points and identify potential outliers. Pearson's product–moment correlation coefficient (r) and Spearman's rank correlation coefficient (ρ), with the latter being more robust to outliers, were calculated to examine discrepancies between the two indices. Potential outliers for each variable were identified using the Smirnov–Grubbs test. Cases identified as outliers were removed from the dataset, and correlation coefficients were recalculated to quantify the impact of outliers on the estimates.

## 3 Results

The characteristics of the 179 patients included in this study are presented in Table 1. Symptomatic vasospasm or DCI occurred in 48 (26.8%) patients. Of the 48 patients, 16 showed cerebral infarctions on CT. The outcome for the 48 patients with symptomatic vasospasm was an mRS score of 0–2 in 17 (35.4%) patients and an mRS score of 3–6 in 31 (64.6%) patients, whereas the outcome for the 131 patients without symptomatic vasospasm was an mRS score of 0–2 in 61 (46.6%) patients and an mRS score of 3–6 in in 70 (53.4%) patients. In univariate analysis (Table 1), original Fisher scale was significantly associated with symptomatic vasospasm. However, age, sex, aneurysm location, Hunt and Kosnic grade, World Federation of Neurosurgical Societies grade, and type of aneurysm treatment did not show this relationship.

### 3.1 Differences in HU values and MEP amplitude correlations with and without DCI

MEPs were successfully recorded in both hands in all 179 patients with SAH.

A representative case is shown in Figures 1A, B: the MEP amplitude (left FDI, 545 μV) of the hemisphere with the higher HU value of the Sylvian fissure (right HU value, 70.37) was higher than that of the contralateral side (left HU value, 35.18; right MEP amplitude, 267 μV). Control MEP reproducibility was confirmed by recording serially every 1 min (purple: first recording, blue: second recording, green: third recording, respectively). None of the patients experienced a decrease in the MEP amplitude during the clipping procedure or exacerbation of postoperative neurological symptoms.

**Figure 2:**
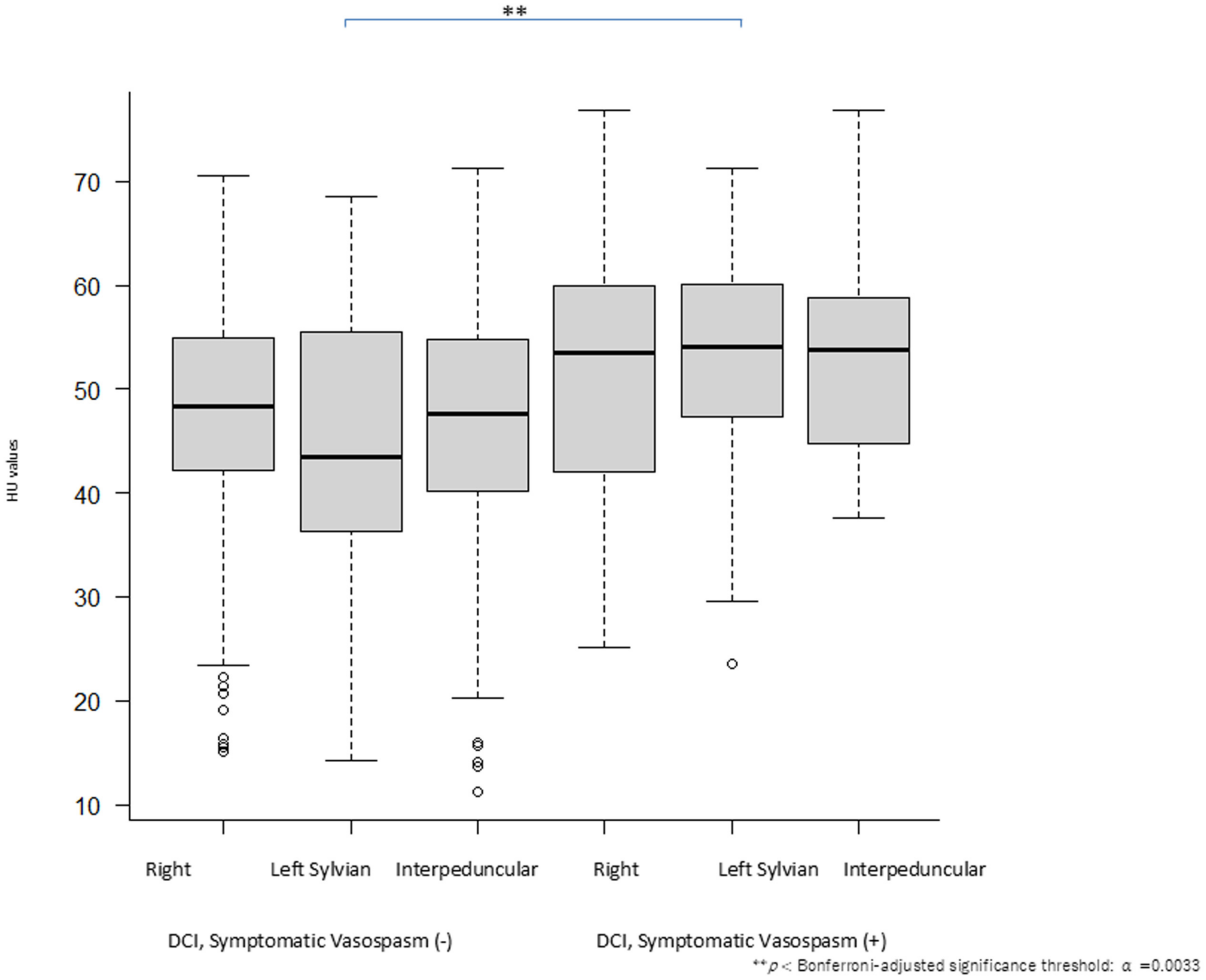
Validations of Hounsfield unit (HU) values: comparison of the right and left Sylvian fissures and the interpeduncular cistern in patients with or without delayed cerebral ischemia (DCI). A multiple comparison test revealed significant differences in the HU values (*p* < Bonferroni-adjusted significance threshold: α = 0.00033). Quantitative differences in HU values were compared among the three locations (right and left Sylvian fissures and the interpeduncular cistern) in patients with and without DCI. Significant differences in HU values were observed in patients with SAH between DCI and non-DCI cases.

The preoperative CT ROI HU values of the bilateral Sylvian fissures and interpeduncular cisterns with and without perioperative DCI were compared. One-way ANOVA revealed that significant difference in HU values [*F*_(5, 531)_ = 5.24, *p* = 0.00014, η^2^ = 0.047; corresponding to Cohen's f = 0.22. These results indicate a small-to-medium effect size and demonstrate significant differences among the groups. Figure 2, Table 2]. The *post-hoc* analysis using Bonferroni method revealed that significant difference in HU values between the DCI (−)-Left Sylvian and DCI (+) Left Sylvian groups (*p* = 0.00211). Effect sizes were calculated as Cohen's d using the pooled standard deviation. Significant contrasts demonstrated medium effect sizes (d = 0.62). These findings indicate that the observed group differences were not only statistically significant but also substantial in magnitude.

**Table 2:** Hounsfield unit and electrophysiological parameters of SAH patients.

	Total	Non-DCI	DCI	*F*	*P-*value
	179	131	48		
HU		46.05 ± 1.14	52.4 ± 1.05	*F*_(5, 531_) = 5.24	< 0.0033
HU Rt.		48.4 ± 11.9	53.5 ± 11.8		
HU IP.		47.6 ± 12.9	53.8 ± 10.1		
HU Lt.		43.4 ± 12.7	54.5 ± 10.5		
MEP					
Threshold (mA)				*F*_(3, 314_) = 0.007	0.91
Rt. motor cortex		59.7 ± 14.7	60.1 ± 11.8		
Lt. motor cortex		59.5 ± 17.6	59.4 ± 12.5		
Amplitude (μV)				*F*_(3, 314_) = 2.99	0.031
Rt. motor cortex		496.6 ± 573.1	588.6 ± 329.8		
Lt. motor cortex		482.5 ± 487.5	721.9 ± 486.9		
RMT (%), TMS-MEP amplitude					
RMT (%)				*F*_(3, 314_) = 1.47	0.22
Rt. motor cortex		52.7 ± 20.2	55.2 ± 14.4		
Lt. motor cortex		50.2 ± 9.7	49.8 ± 8.8		
TMS-MEP amplitude (μV)				*F*_(3, 314_) = 0.81	0.78
Rt. motor cortex		801.5 ± 930.3	908.1 ± 486.5		
Lt. motor cortex		956.5 ± 527.2	956.5 ± 527.3		
SICI		1.24 ± 0.82	1.75 ± 1.58		
				*F*_(4, 373_) = 22.3	< 0.005
Rt. motor cortex		1.66 ± 0.47	2.21 ± 1.97		
Lt. motor cortex		0.82 ± 0.54	1.27 ± 0.85		
ICF		2.64 ± 1.75	1.92 ± 0.88		
				*F*_(4, 373_) = 8.59	< 0.005
Rt. motor cortex		2.87 ± 1.75	1.91 ± 0.86		
Lt. motor cortex		2.42 ± 0.94	1.94 ±0.91		

ICF, intracortical facilitation; RMT, resting motor threshold; SICI, short intracortical inhibition; HU, Hounsfield unit (Rt, Right; Lt, Left Sylvian and IP, Interpeduncular cistern); MEP, motor evoked potential.

MEP control waveform thresholds and amplitudes immediately before surgery are presented in Table 2.

**Figure 3:**
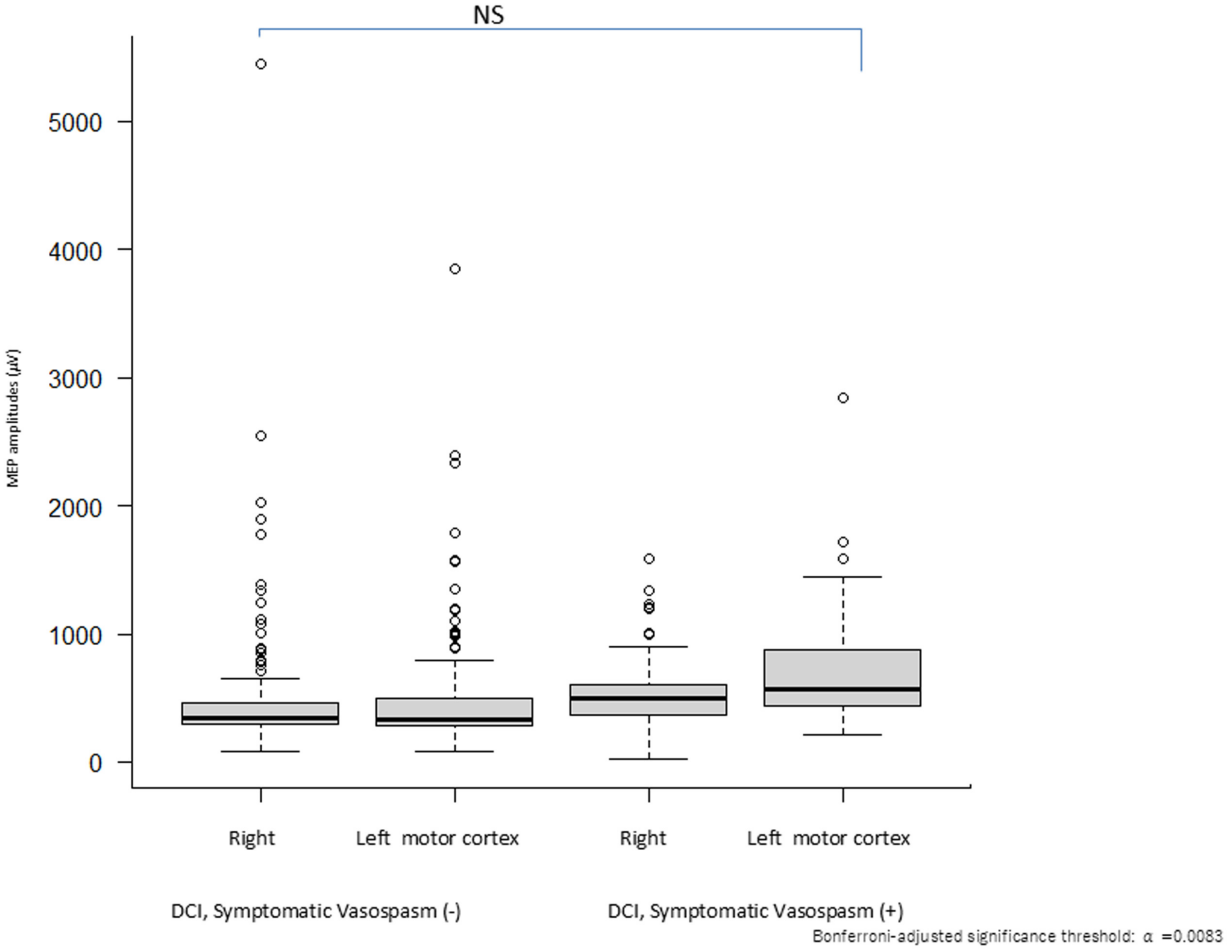
Validations of first dorsal interosseous motor-evoked potential (MEP) amplitudes: comparison between the right and left motor cortices in patients with or without delayed cerebral ischemia (DCI). A multiple comparison test revealed no significant differences in the MEP amplitudes (*p* > Bonferroni-adjusted significance threshold: α = 0.0083). Quantitative differences in MEP amplitudes between the right and left Sylvian fissures with and without DCI were compared between patients with SAH. Significant differences in MEP amplitudes were observed in patients with SAH between DCI and non-DCI cases.

The MEP parameters of the control waveform immediately before surgery were compared with and without DCI in the perioperative period between groups. Electrical stimulation thresholds and MEP amplitudes before surgery did not differ between groups with and without DCI [*F*_(3, 314)_ = 0.007, *p* = 0.9, η^2^ = 6.6e^−05^; *F*_(3, 314)_ = 2.99, *p* = 0.031, η^2^ = 0.025, respectively, Figure 3].

**Figure 4:**
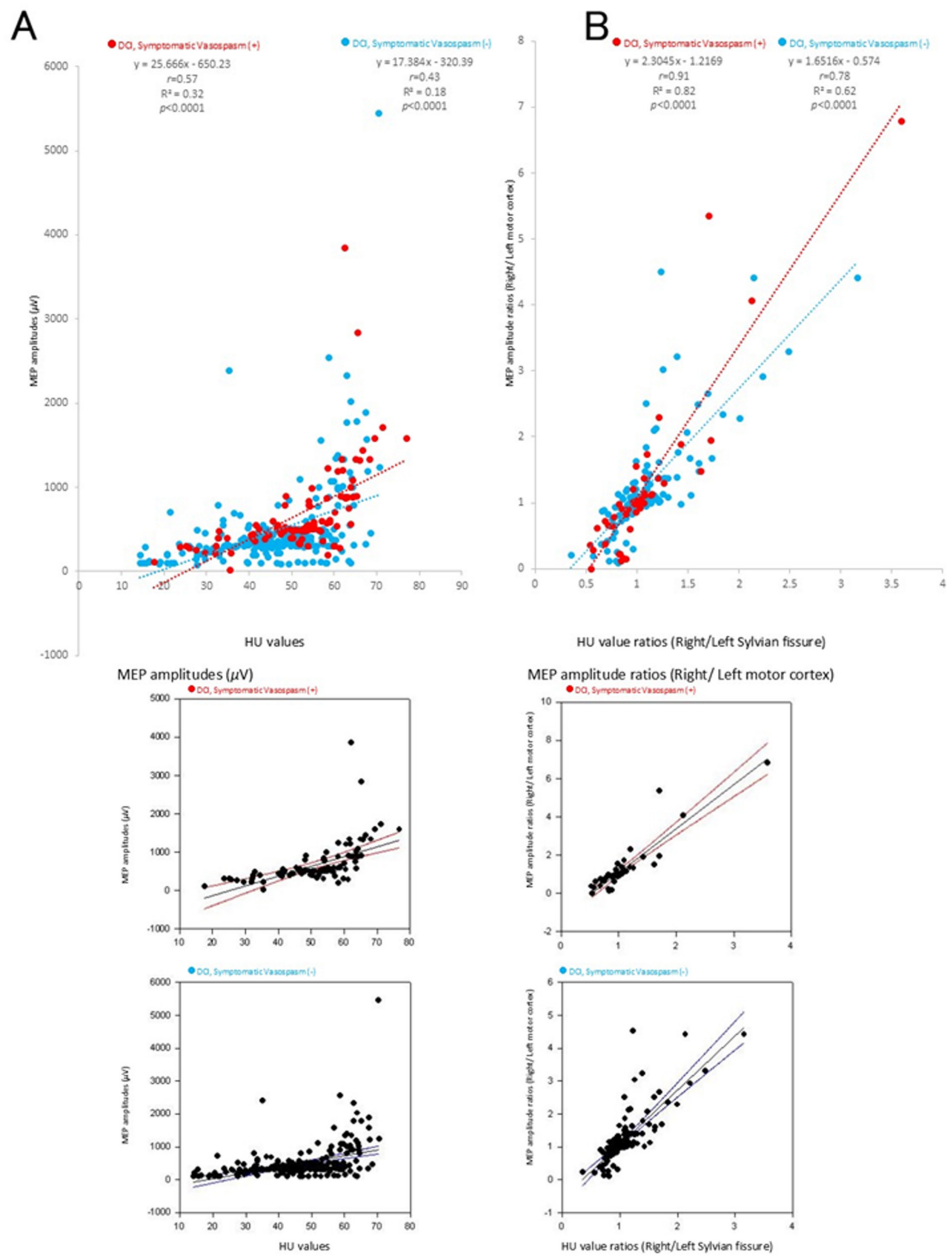
Correlation between Hounsfield unit (HU) values and motor-evoked potential (MEP) amplitudes. Significant correlations were observed between HU values and MEP amplitudes **(A)** and between HU value ratios (right/left Sylvian fissure) and MEP amplitude ratios (right/left motor cortex) **(B)** (*p* < 0.0001). The colors in the graph represent each condition after subarachnoid hemorrhage (SAH) [red: for cases with delayed cerebral ischemia (DCI) or symptomatic vasospasm, blue: for cases without DCI or symptomatic vasospasm in patients with SAH]. The 95% confidence interval (CI) of each regression line are indicated below A and B, respectively.

The MEPs immediately before surgery for the ROI HU values of the bilateral Sylvian fissures showed a significant linear correlation in both groups with and without DCI (*r* = 0.57, R^2^ = 0.32, *p* < 0.0001, red dotted lines and *r* = 0.43, R^2^ = 0.18, *p* < 0.0001, blue dotted lines, respectively; Figure 4A). The MEP ratio (right/left motor cortex) just before surgery to the ROI HU value ratio (right/left) for bilateral Sylvian fissures had a significant linear correlation in both groups with and without DCI (*r* = 0.91, R^2^ = 0.82, *p* < 0.0001, red dotted lines *r* = 0.78, R^2^ = 0.62, blue dotted lines, *p* < 0.0001, Figure 4B). The 95% confidence interval (CI) of each regression line are indicated below Figures 4A, B, respectively. Visual inspection of the scatterplots revealed several potential outliers. Based on all 179 observations, Pearson's correlation coefficient was *r* = 0.57, whereas Spearman's rank correlation coefficient was ρ = 0.74 in the HU-MEP-DCI (+) group (red dots in Figure 4A), indicating a moderate discrepancy between the two measures. The Smirnov–Grubbs test identified two observations as outliers, which were subsequently removed. After excluding these cases, Pearson's correlation decreased to *r* = 0.70, demonstrating that the original estimate was moderately influenced by the outliers. In contrast, Spearman's correlation remained relatively stable (ρ = 0.730), consistent with its robustness to extreme values.

### 3.2 Differences in MEP amplitude and cortical excitability: short-interval intracortical inhibition and intracortical facilitation correlations with and without DCI

**Figure 5:**
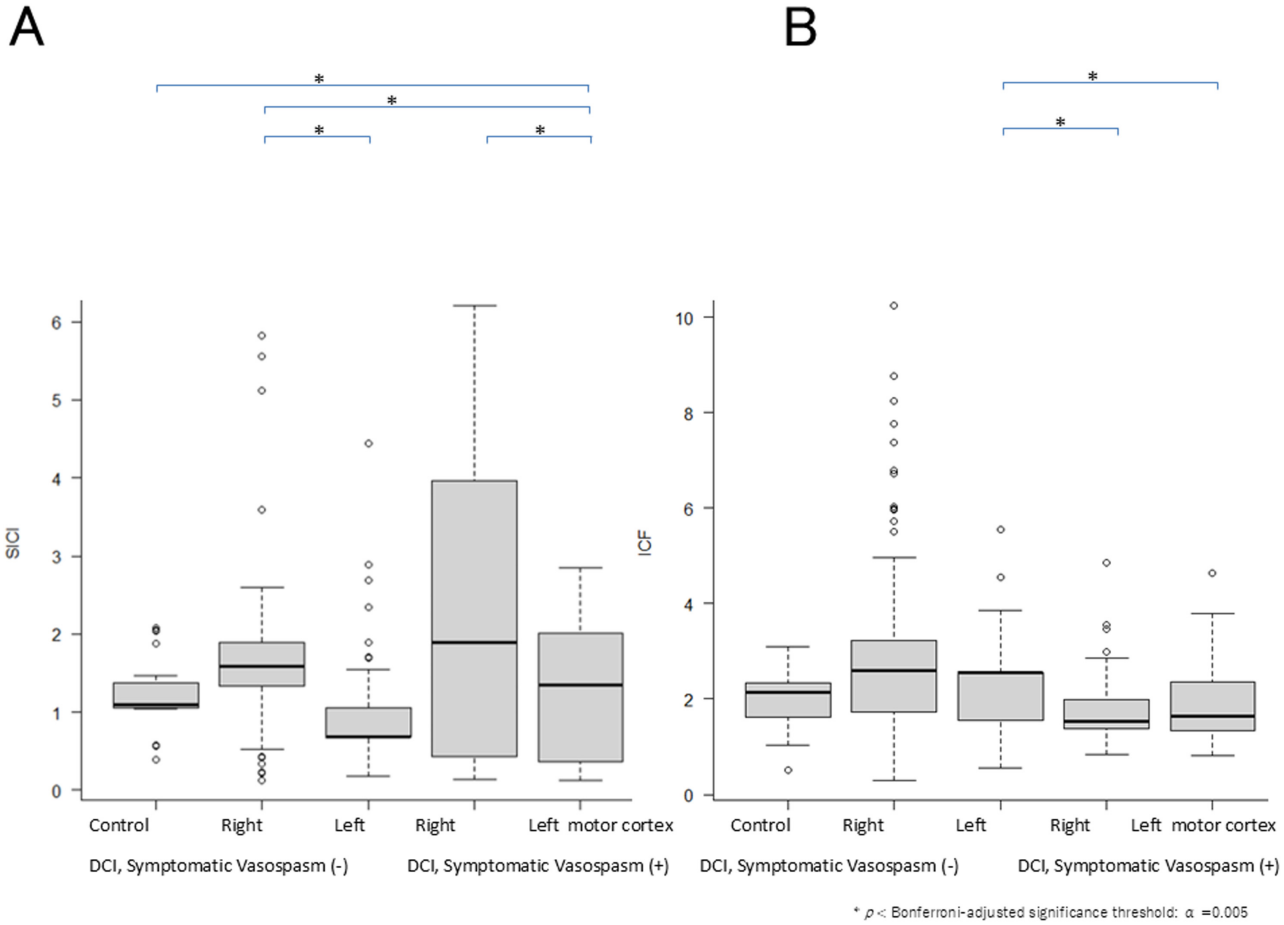
Validations of short-interval intracortical inhibition (SICI) and intracortical facilitation (ICF) values defined from first dorsal interosseous motor-evoked potential: comparison of the right and left motor cortices in healthy controls and patients with subarachnoid hemorrhage (SAH) with or without delayed cerebral ischemia (DCI). A multiple comparison test revealed significant differences in SICI values **(A)** and ICF values **(B)** (*p* < Bonferroni-adjusted significance threshold: α = 0.005). Quantitative differences in SICI and ICF values were compared between the right and left Sylvian fissures in patients with SAH and healthy controls with and without DCI. Significant differences in SICI and ICF were observed in patients with SAH between the DCI and non-DCI groups.

There were no differences in the TMS-MEP parameters, RMT or amplitudes between the control group and the patient groups with and without DCI [RMT: *F*_(5, 332)_ = 0.93, *p* = 0.46, η^2^ = 0.014; Amplitude: *F*_(5, 332)_ = 0.53, *p* = 0.75, η^2^ = 0.007, respectively]. No quantitative differences in the TMS-MEP parameters were observed between the two hemispheres of healthy participants (RMT: 53.2 vs. 52.1%; amplitude: 1,063 vs. 964 μV; SICI: 1.18 vs. 1.25; ICF: 2.08 vs. 1.91, respectively). Additionally, no statistically significant effects of cerebral hemisphere laterality were found for RMT [*t*_(9)_ = 1.46; *P* > 0.05], amplitude [*t*_(9)_ = −0.03; *P* > 0.05), SICI [*t*_(9)_ = −0.31; *P* > 0.05] or ICF [*t*_(9)_ = 0.62; *P* > 0.05; paired *t*-test]. Effect sizes (Cohen's dz) were all < 0.44, indicating negligible-to-small hemispheric differences across measures. Therefore, the average values for both sides were used in the control group: 52.81 ± 9.81%, 1,004.5 ± 543.1 μV, 1.21 ± 0.38, and 2.039 ± 0.59, respectively. Cortical excitabilities, SICI, and ICF, were compared between patients with SAH both with and without perioperative DCI. One-way ANOVA revealed a significant difference in SICI and ICF [*F*_(4, 373)_ = 22.3, *p* = 2e^−16^, η^2^ = 0.238; *F*_(4, 373)_ = 8.59, *p* = 0.0000014, η^2^ = 0.091, Figure 5 and Table 2]. These results indicated a small-to-medium effect size and demonstrated significant differences among the groups. The *post-hoc* analysis using Bonferroni method revealed significant differences in SICI values between the DCI (+)-Left Sylvian and control groups, the DCI (−)-Right Sylvian and DCI (−)-Left Sylvian groups, the DCI (−)-Right Sylvian and DCI (+)-Left Sylvian groups, and the DCI (+)-Right Sylvian and DCI (+)-Left Sylvian groups (*p* = 0.0016, 0.000, 0.000, and 0.00004, respectively). Effect sizes were calculated as Cohen's d using the pooled standard deviations. Significant contrasts demonstrated medium-to-large effect sizes (d = 0.58, 1.18, 1.24, and 0.61, respectively). These findings indicate that the observed group differences were not only statistically significant but also substantial in magnitude.

The *post-hoc* analysis using Bonferroni method revealed significant differences in ICF values between the DCI (+)-Left Sylvian and DCI (−)-Left Sylvian groups and the DCI (+)-Right Sylvian and DCI (−)-Left Sylvian groups (*p* = 0.000067 and 0.00017, respectively). Effect sizes were calculated as Cohen's d using the pooled standard deviations. Significant contrasts demonstrated medium-to-large effect sizes (d = 0.62 and 0.59, respectively). These findings indicate that the observed group differences were not only statistically significant but also substantial in magnitude.

**Figure 6:**
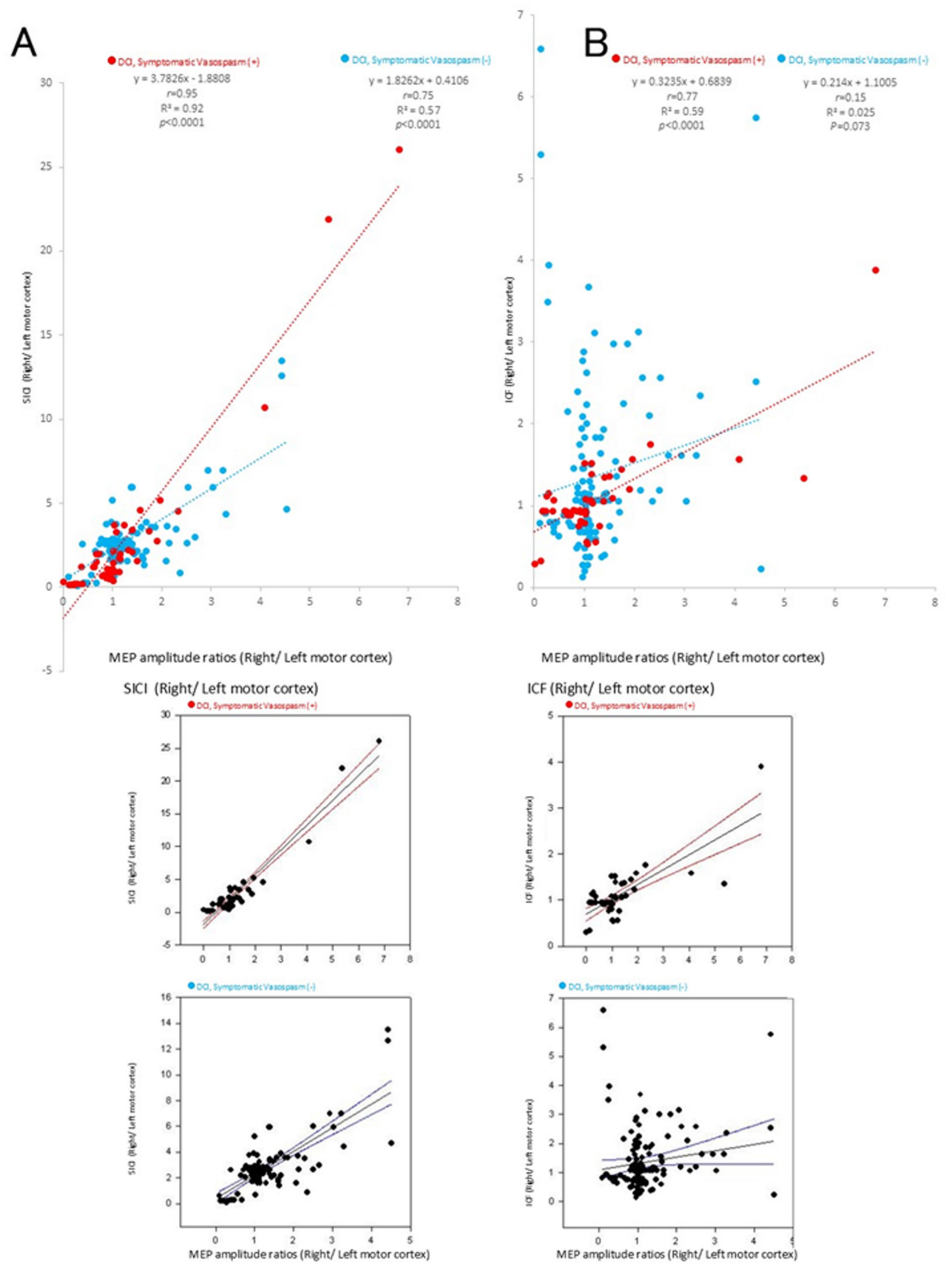
Correlation between motor-evoked potential (MEP) amplitude ratios and short-interval intracortical inhibition (SICI) or intracortical facilitation (ICF). Significant correlations were found between the MEP amplitude ratios and SICI **(A)** and ICF **(B)** (*p* < 0.0001). Significant correlations were observed in the SICI and ICF groups with delayed cerebral ischemia (DCI) (*p* < 0.0001), and a similar trend was observed in the ICF group without DCI; however, this correlation was weak (*p* = 0.073). The colors in the graph represent each condition after subarachnoid hemorrhage (SAH) (red: for cases with DCI or symptomatic vasospasm, blue: for cases without DCI or symptomatic vasospasm in patients with SAH). The 95% confidence interval (CI) of each regression line are indicated below A and B, respectively.

There was a significant linear correlation between the SICI and ICF value ratios (right/left) of the bilateral motor cortex and the MEP ratio (right/left motor cortex) just before surgery in the group with DCI (*r* = 0.95, R^2^ = 0.92, *p* < 0.0001 and *r* = 0.77, R^2^ = 0.59, *p* < 0.0001, respectively; Figures 6A, B, red dotted lines). The SICI in the group without DCI showed a similar trend, although the correlation in ICF was not significant (*r* = 0.75, R^2^ = 0.57, *p* < 0.0001 and *r* = 0.15, R^2^ = 0.025, *p* = 0.073, respectively, Figures 6A, B, blue dotted lines). The 95% CI of each regression line are indicated below Figures 6A, B, respectively. Visual inspection of the scatterplots revealed several potential outliers. Based on all 179 observations, Pearson's correlation coefficient was *r* = 0.76, whereas Spearman's rank correlation coefficient was ρ = 0.45 in the SICI-MEP-DCI (-) group (blue dots in Figure 6A), indicating a moderate discrepancy between the two measures. The Smirnov–Grubbs test identified eleven observations as outliers. After excluding these cases, Pearson's correlation decreased to *r* = 0.53, demonstrating that the original estimate was moderately influenced by the outliers. In contrast, Spearman's correlation remained relatively stable (ρ = 0.34). In addition, Pearson's correlation coefficient was *r* = 0.77, whereas Spearman's rank correlation coefficient was ρ = 0.48 in the ICF-MEP ratio-DCI (+) group (red dots in Figure 6B), indicating a moderate discrepancy between the two measures. The Smirnov–Grubbs test identified four observations as outliers. After excluding these cases, Pearson's correlation decreased to *r* = 0.57, demonstrating that the original estimate was moderately influenced by the outliers. In contrast, Spearman's correlation remained relatively stable (ρ = 0.38).

## 4 Discussion

Intraoperative MEP monitoring during cerebral aneurysm surgery can help avoid major complications, such as infarction due to occlusion of the main artery and perforators (2). Corticospinal tract neuronal damage significantly correlated with a decrease in the intraoperative MEP amplitude. Therefore, surgical procedures should be performed while avoiding a decrease in MEP amplitude of >50%, as these are directly associated with motor impairment (1–3). This study focused on MEP amplitude prior to intraoperative clipping immediately before craniotomy or coil embolization. The MEP amplitude was found to be more strongly facilitated in the hemisphere where SAH was more significant. Furthermore, this study showed that the MEP amplitude facilitation significantly and linearly correlated with the HU value of the initial CT scan. The MEP amplitude facilitation ratio showed a linear correlation with abnormalities in SICI and ICF.

### 4.1 MEP facilitations immediately after SAH correlate with intracortical inhibitory and excitatory interneuronal dysfunction

MEP amplitudes from the bilateral FDI muscles showed a strong linear correlation with the HU values of the bilateral Sylvian fissures in both the DCI and non-DCI groups in the present study. Furthermore, the significantly higher facilitated MEP amplitudes in the DCI group suggest dominant abnormal cortical excitability in the cerebral cortex after SAH.

SICI showed a strong linear correlation with MEP amplitude values in both groups with and without DCI (*r* = 0.95 and *r* = 0.75, respectively), whereas ICF showed a significant linear correlation in the DCI group alone and a similar, albeit weaker (*r* = 0.77 and *r* = 0.15, respectively), correlation in the non-DCI group. Of note, the original estimate was moderately affected by outliers (Figures 4, 6), calling for careful interpretation. Intracortical inhibitory and excitatory neuronal dysfunction may facilitate MEP following SAH. SICI abnormalities account for neuronal dysfunction related to GABA_A_ receptors, which are ligand-gated ion channels (17–19). It is conceivable that the significantly higher SICI values in the DCI group reflect greater damages in the cortical circuits. Our results, showing higher specificity than RMT or MEP (*p* = 2e^−16^ < 0.005), support previous reports (13).

ICF abnormalities are associated with glutamate-mediated excitatory interneuronal function (20).

It is conceivable that ICF facilitation is modulated by the rapid elevation of excitatory glutamate concentrations after SAH (21). Furthermore, increased activity of glutamatergic interneurons within the cortex, distinct from SICI, may also contribute to intracortical facilitation (13, 15). ICF strongly correlates with excitatory glutamatergic interneurons within the motor cortex depending on NMDA receptor activation (16). Interestingly, quadripulse stimulation with a 5-ms interstimulus interval enhances ICF without altering RMT and induces long-term facilitation of TMS-MEP amplitude that is identical to that observed post-SAH (22).

As significant MEP facilitation in the SAH-dominant hemisphere is unexpected, the correlations between MEP amplitudes and HU values found in our study may be associated with motor neuronal and intracortical excitatory and/or inhibitory interneuronal dysfunctions caused by SAH and an interhemispheric balance between excitatory and inhibitory circuits (13).

### 4.2 MEP facilitation could indicate a motor cortical ischemic state

Conversely, cases have been reported in which the amplitude of the MEP increases after surgery, followed by a rapid decrease and disappearance of the amplitude (23–25). It is speculated that this is due to differences in the ischemia tolerance of pyramidal cells and inhibitory interneurons: first, amplitude increases as inhibition is removed; then, it subsequently decreases and disappears due to pyramidal cell ischemia (24). Transient increases in the excitability of cortical motor and hippocampal neurons in the early stages of ischemia have been confirmed experimentally (26–29), which is consistent with the increased MEP amplitude during surgical procedures involving temporary proximal trunk artery blockade.

GABA-A receptors are prone to internalization and degradation during ischemia, which makes the entire GABAergic system susceptible to early impairment (26). Dysfunction or loss of GABAergic neurons induces reduced inhibition, beginning with diminished SICI. Thus, the fact that the MEP amplitude increases more strongly in the hemisphere where SAH is present can be interpreted as an indicator of an ischemic state of GABAergic inhibitory interneurons. However, it is essential to alert the surgeon not only to a decrease or loss of MEP amplitude associated with surgical maneuvers but also to an increase in MEP amplitude. An increase in MEP amplitude just prior to surgery is likely to be the result that must be fully considered to be the summation of changes in cortical excitability due to SAH itself, increased intracranial pressure, EBI, and microcirculatory disturbance (6).

### 4.3 Limitations and future work

First, the thresholds and other electrophysiological parameters in the MEPs of the patients in the present study may have been affected by their general condition, which is associated with an unusual elevation in intracranial pressure and blood pressure during the acute stage of stroke. Furthermore, FDI-MEP only reflects a portion of the function of the cortico-hand muscle group and is not always representative of whole-brain function. Careful interpretation is required, as whole-brain neuronal excitability may differ from corticospinal FDI-MEP parameters and perioperative neurological functions, both of which are non-linear. Moreover, since propofol induces GABA-A receptor-mediated SICI enhancement, ICF reduction, RMT elevation, and amplitude reduction (16), it should be considered as a confounding factor in the obtained results.

Second, the number of participants in the age-matched control group was significantly smaller compared with that in the patient group. Furthermore, the MEP 140/120 ratio, a well-distinguished parameter of cortical excitability (13, 15), failed to provide an assessment. This could affect the influence of handedness. If neuronal transmission is more efficient in the dominant hemisphere, the MEP amplitude on the dominant hemisphere (left side) may be reduced. Comparing the SAH group to the control group may provide new insight. The present results found no differences in the TMS-MEP parameters between the control group and the patient groups. However, it cannot be denied that the MEP amplitude ratio (right/left motor cortex) may be influenced by the difference in motor threshold between the left and right motor cortices. Furthermore, in healthy individuals, the dominant hemisphere is considered to have slightly higher excitability than the contralateral hemisphere (bilateral differences and ratios are approximately RMT: 3–5%, 0.95–1.05; SISI: nearly symmetrical, 0.8–1.2; ICF: nearly symmetrical, 0.8–1.2) (15, 30). In stroke patients, the stronger contralateral primary motor cortex may excessively inhibit the weakened ipsilateral primary motor cortex (interhemispheric inhibition [IHI]; enhanced IHI), leading to overall network asymmetry (30).

Interpeduncular HU values in the initial CT scan (45.3% above 60) are accurate and reliable predictors of symptomatic vasospasm (4). Similar observations regarding the use of initial UH values as a predictor of complications during perioperative period of SAH have been reported (7, 8).

Since the advent of clazosentan, the onset of symptomatic vasospasm has been suppressed, changing the postoperative management of SAH and its prognosis (31, 32) and making it difficult to apply the HU value criterion alone.

Notably, many patients with SAH with HU values >60 avoid DCI in a recent trend (2).

The present study revealed a significant positive linear correlation between the MEP high-amplitude ratio and the Sylvian fissure HU value ratio and a trend toward the development of DCI onset. Further case studies are required to establish new indicators, particularly given the increasing diversity in the postoperative management of SAH. Combining SICI/ICF and IHI with HU/MEP values of left-right hemisphere ratios may make predicting DCI onset more reliable than relying solely on HU or MEP values. Simultaneously evaluating the corticospinal tract output via MEP and RMT, intracortical circuits state via SICI and ICF, and interhemispheric connections via IHI enables a comprehensive assessment of lateral differences in the motor cortex network. This approach can identify of disruptions in interhemispheric balance and asymmetries in plasticity that were previously difficult to capture with single indicators. Thus, it deepens our understanding of the neurophysiological mechanisms underlying motor function. Simultaneously and longitudinally evaluating RMT, MEP amplitude, SICI, ICF and interhemispheric connectivity via IHI provides a comprehensive view of brain networks and could allow for more detailed, real-time prognostic predictions. Above all, the greatest advantage of this method is its non-invasive implementation at the bedside without requiring patient transfer. Tracking changes in cortical excitability and inhibition over time using neurophysiological techniques and postoperative TMS (11, 12, 16) may enable a more non-invasive and detailed assessment of ongoing pathology after SAH.

## 5 Conclusion

The present study explored that the MEP amplitude in the hemisphere with more significant SAH was significantly higher than that in the contralateral hemisphere. This difference linearly correlated with HU and abnormalities in SICI and ICF (*p* < 0.0001). High MEP amplitudes just before surgery correlate well with Sylvian fissure HU values on initial CT and may reflect SAH cortical electrophysiological intracortical inhibitory–facilitatory neural circuit dysfunction. Simultaneous and longitudinal evaluation of combined RMT, MEP amplitude, SICI, ICF, and IHI could be potential real-time predictors of DCI.

## Data Availability

The original contributions presented in the study are included in the article/supplementary material, further inquiries can be directed to the corresponding author.

## References

[1] Suzuki K Kodama N Sasaki T Matsumoto M Konno Y Sakuma J . Intraoperative monitoring of blood flow insufficiency in the anterior choroidal artery during aneurysm surgery. J Neurosurg. (2003) 98:507–14. doi: 10.3171/jns.2003.98.3.0507 12650421

[2] Sasaki T Kodama N Matsumoto M Suzuki K Konno Y Sakuma J . Blood flow disturbance in perforating arteries attributable to aneurysm surgery. J Neurosurg. (2007) 107:60–7. doi: 10.3171/JNS-07/07/0060 17639875

[3] Fujiki M Furukawa Y Kamida T Anan M Inoue R Abe T . Intraoperative corticomuscular motor evoked potentials for evaluation of motor function: a comparison with corticospinal D and I waves. J Neurosurg. (2006) 104:85–92. doi: 10.3171/jns.2006.104.1.85 16509151

[4] Ishihara H Oka F Kawano R Shinoyama M Nishimoto T Kudomi S . Hounsfield unit value of interpeduncular cistern hematomas can predict symptomatic vasospasm. Stroke. (2020) 51:143–8. doi: 10.1161/STROKEAHA.119.026962 31694506

[5] Suzuki H Kanamaru H Kawakita F Asada R Fujimoto M Shiba M. Cerebrovascular pathophysiology of delayed cerebral ischemia after aneurysmal subarachnoid hemorrhage. Histol Histopathol. (2021) 36:143–58. doi: 10.14670/HH-18-253 32996580

[6] Dreier JP. The role of spreading depression, spreading depolarization and spreading ischemia in neurological disease. Nat Med. (2011) 17:439–47. doi: 10.1038/nm.2333 21475241

[7] Nomura Y Kawaguchi M Yoshitani K Kurita N Hayashi H Tamura K . Retrospective analysis of predictors of cerebral vasospasm after ruptured cerebral aneurysm surgery: influence of the location of subarachnoid blood. J Anesth. (2010) 24:1–6. doi: 10.1007/s00540-009-0836-2 20039076

[8] Park JS Kang HG. Hounsfield unit as a predictor of symptomatic vasospasm and hydrocephalus in good-grade subarachnoid hemorrhage treated with endovascular coiling. Quant Imaging Med Surg. (2023) 13:6627–35. doi: 10.21037/qims-23-355 37869270 PMC10585502

[9] Herwig U Schönfeldt-Lecuona C Wunderlich AP Von Tiesenhausen C Thielscher A Walter H . The navigation of transcranial magnetic stimulation. Psychiatry Res. (2001) 108:123–31. doi: 10.1016/S0925-4927(01)00121-4 11738546

[10] Lefaucheur JP. Why image-guided navigation becomes essential in the practice of transcranial magnetic stimulation. Neurophysiol Clin. (2010) 40:1–5. doi: 10.1016/j.neucli.2009.10.004 20230930

[11] Fujiki M Matsushita W Kawasaki Y Fudaba H. Monophasic-quadripulse theta burst magnetic stimulation for motor palsy functional evaluation after intracerebral hemorrhage. Front Integr Neurosci. (2022) 16:827518. doi: 10.3389/fnint.2022.827518 35359705 PMC8963344

[12] Fujiki M Hata N Anan M Matsushita W Kawasaki Y Fudaba H. Monophasic-quadri-burst stimulation robustly activates bilateral swallowing motor cortices. Front Neurosci. (2023) 17:1163779. doi: 10.3389/fnins.2023.1163779 37304027 PMC10248141

[13] Neville IS Gomes Dos Santos A Almeida CC Hayashi CY Solla DJF Galhardoni R . Evaluation of changes in preoperative cortical excitability by navigated transcranial magnetic stimulation in patients with brain tumor. Front Neurol. (2021) 11:582262. doi: 10.3389/fneur.2020.582262 33551949 PMC7863982

[14] Hayashi CY Neville IS Rodrigues PA Galhardoni R Brunoni AR Zaninotto AL . Altered intracortical inhibition in chronic traumatic diffuse axonal injury. Front Neurol. (2018) 9:1–8. doi: 10.3389/fneur.2018.00189 29643831 PMC5882787

[15] Cueva AS Galhardoni R Cury RG Parravano DC Correa G Araujo H . Normative data of cortical excitability measurements obtained by transcranial magnetic stimulation in healthy subjects. Neurophysiol Clin. (2016) 46:43–51. doi: 10.1016/j.neucli.2015.12.003 26924308

[16] Fujiki M Kuga K Ozaki H Kawasaki Y Fudaba H. Blockade of motor cortical long-term potentiation induction by glutamatergic dysfunction causes abnormal neurobehavior in an experimental subarachnoid hemorrhage model. Front Neural Circuits. (2021) 15:670189. doi: 10.3389/fncir.2021.670189 33897380 PMC8063030

[17] Nakamura H Kitagawa H Kawaguchi Y Tsuji H. Intracortical facilitation and inhibition after transcranial magnetic stimulation in conscious humans. J Physiol. (1997) 498:817–23. doi: 10.1113/jphysiol.1997.sp021905 9051592 PMC1159197

[18] Keller A. Intrinsic synaptic organization of the motor cortex. Cereb Cortex. (1993) 3:430–41. doi: 10.1093/cercor/3.5.430 8260811

[19] Cash RFH Noda Y Zomorrodi R Radhu N Farzan F Rajji TK . Characterization of glutamatergic and GABA A-mediated neurotransmission in motor and dorsolateral prefrontal cortex using paired-pulse TMS-EEG. Neuropsychopharmacology. (2017) 42:502–11. doi: 10.1038/npp.2016.133 27461082 PMC5399228

[20] Chen R. Interactions between inhibitory and excitatory circuits in the human motor cortex. Exp Brain Res. (2004) 154:1–10. doi: 10.1007/s00221-003-1684-1 14579004

[21] Kawakita F Kanamaru H Asada R Suzuki Y Nampei M Nakajima H . Roles of glutamate in brain injuries after subarachnoid hemorrhage. Histol Histopathol. (2022) 37:1041–51. doi: 10.14670/HH-18-509 36065974

[22] Hamada M Terao Y Hanajima R Shirota Y Nakatani-Enomoto S Furubayashi T . Bidirectional long-term motor cortical plasticity and metaplasticity induced by quadripulse transcranial magnetic stimulation. J Physiol. (2008) 586:3927–47. doi: 10.1113/jphysiol.2008.152793 18599542 PMC2538917

[23] Horiuchi K Suzuki K Sasaki T Matsumoto M Sakuma J Konno Y . Intraoperative monitoring of blood flow insufficiency during surgery of middle cerebral artery aneurysms. J Neurosurg. (2005) 103:275–83. doi: 10.3171/jns.2005.103.2.0275 16175857

[24] Gurung P Motoyama Y Takatani T Takamura Y Takeshima Y Matsuda R . Transient augmentation of intraoperative motor evoked potentials during middle cerebral artery aneurysm surgery. World Neurosurg. (2019) 130:e127–32. doi: 10.1016/j.wneu.2019.06.004 31201943

[25] Maruta Y Fujii M Imoto H Nomura S Tanaka N Inamura A . Strategies and pitfalls of motor-evoked potential monitoring during supratentorial aneurysm surgery. J Stroke Cerebrovasc Dis. (2016) 25:484–95. doi: 10.1016/j.jstrokecerebrovasdis.2015.10.025 26639401

[26] Bolay H Gürsoy-Ozdemir Y Unal I Dalkara T. Altered mechanisms of motor-evoked potential generation after transient focal cerebral ischemia in the rat: implications for transcranial magnetic stimulation. Brain Res. (2000) 873:26–33. doi: 10.1016/S0006-8993(00)02466-5 10915807

[27] Howard EM Gao TM Pulsinelli WA Xu ZC. Electrophysiological changes of CA3 neurons and dentate granule cells following transient forebrain ischemia. Brain Res. (1998) 798:109–18. doi: 10.1016/S0006-8993(98)00403-X 9666096

[28] Luhmann HJ Heinemann U. Hypoxia-induced functional alterations in adult rat neocortex. J Neurophysiol. (1992) 67:798–811. doi: 10.1152/jn.1992.67.4.798 1316953

[29] Luhmann HJ Mudrick-Donnon LA Mittmann T Heinemann U. Ischaemia-induced long-term hyperexcitability in rat neocortex. Eur J Neurosci. (1995) 7:180–91. doi: 10.1111/j.1460-9568.1995.tb01054.x 7538854

[30] Murase N Duque J Mazzocchio R Cohen LG. Influence of interhemispheric interactions on motor function in chronic stroke. Ann Neurol. (2004) 55:400–9. doi: 10.1002/ana.10848 14991818

[31] Endo H Hagihara Y Kimura N Takizawa K Niizuma K Togo O . Effects of clazosentan on cerebral vasospasm-related morbidity and all-cause mortality after aneurysmal subarachnoid hemorrhage: two randomized phase 3 trials in Japanese patients. J Neurosurg. (2022) 137:1707–17. doi: 10.3171/2022.2.JNS212914 35364589

[32] Pontes JPM Santos MDC Gibram FC Rodrigues NMV Cavalcante-Neto JF Barros ADM . Efficacy and safety of clazosentan after aneurysmal subarachnoid hemorrhage: an updated meta-analysis. Neurosurgery. (2023) 93:1208–19. doi: 10.1227/neu.0000000000002601 37462365

